# High throughput data analyses of the immune characteristics of *Microtus fortis* infected with *Schistosoma japonicum*

**DOI:** 10.1038/s41598-017-11532-2

**Published:** 2017-09-12

**Authors:** Yuan Hu, Lei Sun, Zhongying Yuan, Yuxin Xu, Jianping Cao

**Affiliations:** National Institute of Parasitic Diseases, Chinese Center for Disease Control and Prevention, Key Laboratory of Parasite and Vector Biology, MOH, China, National Center for International Research on Tropical Diseases, China, WHO Collaborating Center for Tropical Diseases, Shanghai, 200025 China

## Abstract

*Microtus fortis* exhibits natural resistance against *Schistosoma japonicum*, and the parasite cannot grow and develop in *M. fortis*. Extensive research has been carried out, however, the associated mechanism remains unclear. In the present study, we analysed the combined data obtained from a cytokine chip assay, transcriptome, and metabolome. The cytokine profile from C57BL/6 and *M. fortis* mice was assessed before and after infection. Several cytokines increased during the second and third week post-infection. Some transcripts related to cytokine genes and associated proteins were also highly expressed (i.e., *Hgf, C3*, and *Lbp*). The liver metabolism of *M. fortis* following infection with *S. japonicum* was assessed. We identified 25 different metabolites between the uninfected and infected *M. fortis*, and 22 different metabolites between infected *M. fortis* and C57BL/6 mice. The metabolomic pathways of these differential metabolites were then analysed with MetPA, revealing that they were involved in histidine metabolism, valine, leucine, and isoleucine biosyntheses, and lysine degradation. Thus, the elevated expression of these metabolites and pathways may promote the phagocytic function of the neutrophils and natural killer cell activity following TLR activation. These results provide novel insight into the resistance mechanism of *M. fortis* against *S. japonicum*.

## Introduction

Schistosomiasis is primarily caused by three species of parasitic flatworms, *Schistosoma japonicum, S. mansoni*, and *S. haematobium*, which infect approximately 250 million people in 78 countries (Who, February 2014) and result in the loss of 1.53 million disability-adjusted life years (DALYs) annually^1^. *S. japonicum* is highly prevalent in China, Indonesia, and the Philippines and currently endemic in the Anhui, Hubei, Hunan, Jiangxi, Sichuan, and Yunnan provinces in China^2^. Schistosomiasis japonica is caused by lodged schistosome eggs in the host liver, leading to an inflammatory response, hepatic granuloma formation, and fibrosis. *S. japonicum* has a very wide host range, infecting at least 31 species of wild mammals, including permissive hosts (e.g., C57BL/6 mice) and non-permissive hosts (e.g., SD rats and *Microtus fortis*). The characteristics of the immune response also differ between hosts^3^.

*M. fortis* is a non-permissive host of *S. japonicum*, which exhibits a natural resistance against *S. japonicum* infection. It has been reported that the cercaria which invade via the skin, migrate through the lungs to the liver, and eventually die in the livers of *M. fortis* ^4^. Moreover, 12 days post-infection, white inflammatory nodules are observed on the surface of the *M. fortis* liver, which were composed of the complete worm surrounded by a large amount of inflammatory cells^5^. Some reports have presented data from the Dongting Lake region, in which *S. japonicum* is highly prevalent, and a total of 1,440*M. fortis* were captured. Upon examination, there were no eggs, adult worms, or schistosomula identified in the captured *M. fortis* when examined. Therefore, it was concluded that *M. fortis* cannot be infected with *S. japonicum* ^6^. Scientists have performed multiple studies regarding the resistance of *M. fortis* against *S. japonicum*, and many factors, including complement, IgG, albumin, KPNA2, and HSP90 were identified to play a role in resistance^7–11^; however, the specific mechanism by which resistance occurs remains unclear.

The growth and development of schistosome is related to the cytokines produced by the host. Riner’s report demonstrated that neutralizing TNF using anti-TNF antibodies restored parasite development in immunodeficient mice, and the administration of IL-4 to immunodeficient mice regulated inflammation and restored parasite development^12^. Moreover, host metabolism is closely related to its immune function^13^. Thus, metabolomics can reveal the cellular response to *S. japonicum* infection^14^. Additionally, combining the cytokine, transcriptome, and metabolomic profiles may provide novel insight into the resistance mechanism of *M. fortis* against *S. japonicum*.

In this study the immunological characteristics of *M. fortis* infected with *S. japonicum* were analysed using transcriptome, cytokine array, and metabolomic data. This study provides novel insight into the resistance mechanisms employed by *M. fortis* against *S. japonicum*.

## Materials and Methods

### Ethics statement

This study was performed according to the recommendations of the Laboratory of Animal Welfare and Ethics Committee (LAWEC) of China. The protocol was approved by the LAWEC Committee of the National Institute of Parasitic Diseases, Chinese Centre for Disease Control and Prevention (approval ID: IPD 2009–4). All surgery was performed under sodium pentobarbital anaesthesia, and all efforts were made to minimize animal suffering.

### Animal infection and sample collection

A total of 15*M. fortis* and 15 C57BL/6 mice were shaved and cutaneously infected with a mainland strain of *S. japonicum* cercariae. Based on each rodent species’ susceptibility to *S. japonicum*, *M. fortis* was infected with 1,000 cercariae and C57BL/6 mice were infected with 40 cercariae per individual. Sera were collected from the animals either before infection or after the first, second, third, or fourth week post-infection, and stored at −80 °C until further testing. After sacrifice, the livers were removed, frozen in liquid nitrogen, and stored at −80 °C until further use.

Infected *Oncomelanias hupensis* snails were provided by the National Institute of Parasitic Diseases, Chinese Center for Disease Control and Prevention. The infected snails were placed in dechlorinated water under artificial light to induce cercarial shedding prior to infecting the mice. Two weeks post-infection, the animals were intraperitoneally administered 0.75% pentobarbital sodium. After the induction of anaesthesia, the animals were sacrificed. The liver tissues were sterilely removed and frozen at −80 °C.

### Cytokine detection and analysis

The levels of 20 different cytokines were determined using a quantify mouse cytokine (QAM-CYT-1-1) antibody array in accordance with the manufacturer’s instructions (RayBiotech, Norcross, GA). Briefly, forceps were used to handle the membranes, which were gripped by the edges only. Blocking buffer was added and incubated at room temperature for 30 min to block the slide. The blocking buffer was then discarded and the slide was incubated in 100 μL sera at room temperature for 2 h. The samples were diluted with 1× blocking buffer, the sera were discarded, and the slide was washed five times with washing buffer. Next, 80 μL biotin-conjugated antibodies were added to each well and incubated at room temperature for 2 h. The biotin-conjugated antibodies were discarded and washed five times, followed by the addition of 80 μL Cy3-conjugated streptavidin to each well and incubation at room temperature for 1 h. The Cy3-conjugated streptavidin was discarded and washed five times. The washing buffer was then discarded and the plate was centrifuged at 1000 rpm for 3 min to eliminate any excess washing buffer. An Axon GenePix laser scanner was used to scan the signal (excitation frequency = 532 nm; PMT = 650). The fluorescent signal of the original data was then extracted using Genepix 4000B software. The data were analysed by RayBio QAM-CYT-1 analysis software. After the data from all of the samples were averaged, the concentrations of each factor in the samples were calculated according to the associated standard curve. Similar results were observed in an independent array.

### RNA-seq and data analysis

Four independent cDNA libraries were constructed for the four *M. fortis* liver samples according to the RNA-Seq protocol. Raw image files were collected using the Illumina HiSeqTM2000 sequencing platform in BGI Shenzhen, China (http://en.genomics.cn/navigation/index.action). The analyzed data have been deposited in the NCBI Sequence Read Archive under the Accession No. SRX337491.Screening of DEGs was carried out by GO, KEGG analysis^15^.

### Screening of the intersection between transcription and cytokine chip array

Intersection screening was carried out between the cytokines uniport ID and unigene uniport ID for transcription. The String-db database was used to identify proteins that interacted with the 20 cytokines assessed with cytokine chip array. Intersection screening was also performed between the interacted protein and swissport annotation information obtained during transcription.

### Construction of the regulatory model

A gene function enrichment analysis of various cytokines was performed using GO, KEGG and protein-protein-interaction (PPI). Biological function-related terms and signalling pathways with significance were selected to establish a regulatory model using the software, cytoscape 3.1.

### LC-MS and data analysis

A total of 14 *M. fortis* and 14 C57B6 mice were divided into four groups, consisting of an uninfected *M. fortis* (Group A), infected *M. fortis* (Group B), uninfected C57BL/6 mice (Group C) and infected C57BL/6 mice (Group D). The animals were transcutaneously infected with cercariae. Two weeks post-infection, the livers of all animals were detected using liquid chromatography-mass spectrometry (LC-MS).

Fifty-milligram liver samples were collected from each individual, and 1000 μL of solvent (acetonitrile:methanol:water = 2:2:1) was added to the homogenate. Five hundred-microliter tissue homogenate samples were centrifuged at 10 000 g for 10 min at 4 °C. Approximately 400 μL of supernatant was then concentrated to dryness in a TurboVap nitrogen evaporator (Zymax Corp., USA). One hundred microliters of acetonitrile-water (1:1, v/v) solvent was added to redissolve the samples, which were then vortexed for 5 min and centrifuged at 10 000 g for 10 min at 4 °C. Eighty-microlitre samples of the supernatants were used in the LC/MS analyses.

The instrumental platform was a LC-Q/TOF-MS (Agilent, 1290 InfinityLC, 6530 UHD, and Accurate-Mass Q-TOF/MS). The separation columns included a C18 column (Agilent, 100 mm × 2.1 mm, 1.8 μm). The chromatographic separation conditions included a column temperature of 40 °C and a flow rate of 0.35 mL/min. Mobile phase A consisted of water and 0.1% formic acid. Mobile phase B consisted of acetonitrile and 0.1% formic acid. The injection volume was 4 μL, and the temperature of the autosampler was 4 °C.

The raw data were extracted and normalized to the total peak area before performing a multivariate statistical analysis. The retention time, m/z value, and intensity for each sample was detected and reformed into an Excel matrix. The data were imported into the Simca-P software (version 11.5, Umetrics AB, Sweden) and used for principal component analysis (PCA), partial least squares discriminant analysis (PLS-DA), and orthogonal partial least squares discriminant analysis (OPLS-DA). Pareto scaling was used before completing the multivariate analysis, and variable importance in the projection (VIP) was calculated. The differential metabolites were preliminarily identified according to VIP values of the OPLS combined with the *P* value of the Student’s *t-*test using SPSS 11.5.

### Metabolomics pathway analysis

Metabolomics pathway analysis (MetPA) (http://metpa.metabolomics.ca) is a web-based tool that combines the results from powerful pathway enrichment analyses with those of pathway topology analyses to help researchers identify pathways that are the most relevant to the conditions under study. One essential step is the standardization of the compound labels for comparison with the compounds contained in the pathway library. KEGG was used as the database source, and the impact-value threshold was set as 0.10^16^. For our study, after the detection of 28 samples from the four groups, the differential metabolites were analysed to determine which metabolic pathways they belonged to by MetPA. The metabolomic methods and analysis were carried out by Shanghai Sensichip Infotech Co., Ltd (Shanghai, China).

## Results

### Immune activation occurs earlier in *M. fortis*

**Figure 1:**
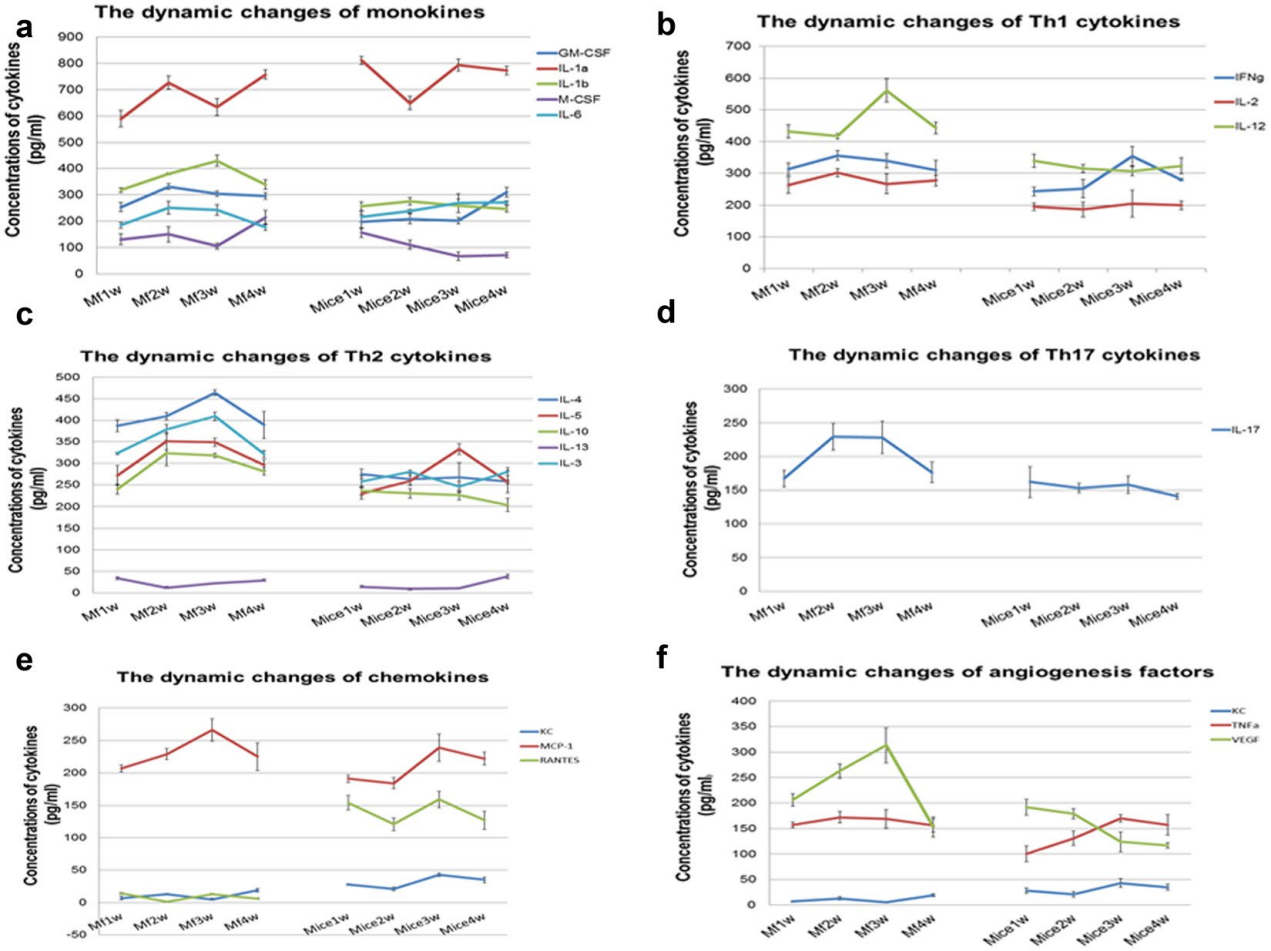
The dynamic cytokine changes exhibited by *M. fortis* and C57BL/6 mice following infection with *S. japonicum*. The cytokine levels in the sera of *M* *. ﻿fortis* were significantly higher from second to third week post-infection compared to that of C57BL/6 mice, including 1a, monokines (e.g., IL-1α, IL-1b, and GM-CSF); 1b, Th1 cytokines (e.g., IL-12, IFN-γ, and IL-2); 1c, Th2 cytokines (e.g., IL-4, IL-5, IL-10, and IL-3); 1d, Th17 cytokine (i.e., IL-17); 1e, chemokines (i.e., MCP-1); 1 f, angiogenesis factors (e.g., VEGF and TNF-α).

In the sera of *M. fortis*, the levels of IL-1b, IL-3, IL-4, IL-10, IL-17, MCP-1 and VGF were found to increase from the second to the third week post-infection. During this period, the cytokine levels did not change significantly in the sera of C57BL/6 mice. In particular, changes in regulated on activation normal T cell expressed and secreted (RANTES) differed between *M. fortis* and C57BL/6 mice. The level of RANTES was lower in *M. fortis* than that of C57BL/6 mice; however, the levels of monokines, Th1, Th2 and Th17 cytokines, chemokines, and angiogenesis were higher in *M. fortis* than that of C57BL/6 mice. Macrophage activation in *M. fortis* occurred earlier than in C57BL/6 mice, while T lymphocytes in C57BL/6 mice might be more active than in *M. fortis* (Fig. 1).

### Cytokine and transcriptome correlation analysis showed that IL-4, KC, TNF-α and several processes in the innate immune response may be important against *S. japonicum* infection in *M. fortis*

**Figure 2:**
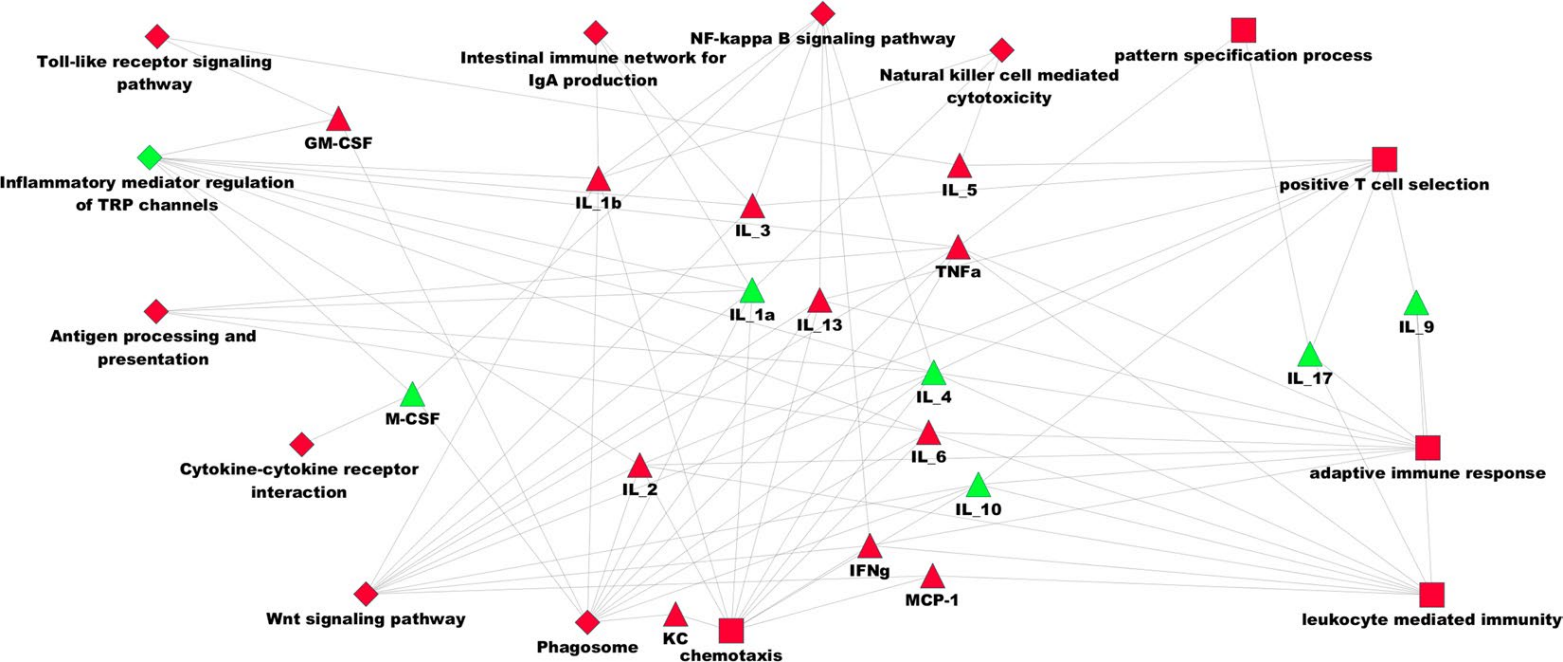
Regulation and interaction network model of 17 cytokines. The triangle represents cytokines. Green indicates down-regulation (Fold change <1), and red represents up-regulation (Fold change >1). The diamond denotes the KEGG signalling pathway, and the rectangle indicates biological processes. Red was significantly expressed (P value < 0.05), and green was not significantly expressed (P value > 0.05).

During the comparison of the cytokines to UniGene sequences through BLASTx, some transcript-related cytokine genes and interacting proteins were highly expressed. There were twelve transcripts associated with seven cytokines, and more than 100 transcripts were associated with 16 cytokine-associated proteins. This indicated that the results of cytokine chip and transcriptome are consistent. These biological processes and signalling pathways (Table 1) were used to construct a regulatory model. According to these terms and information of cytokines and transcriptomes, regulated net was constructed (Fig. 2).

**Table 1:** Selected GO/KEGG terms for constructing the regulatory model.

Term	Source
*Phagosome*	*KEGG*
*Wnt signaling pathway*	*KEGG*
*Inflammatory mediator regulation of TRP channels*	*KEGG*
*Antigen processing and presentation*	*KEGG*
*Intestinal immune network for IgA production*	*KEGG*
*NF-kappa B signaling pathway*	*KEGG*
*Toll-like receptor signaling pathway*	*KEGG*
*Natural killer cell mediated cytotoxicity*	*KEGG*
*Cytokine-cytokine receptor interaction*	*KEGG*
*TNF signaling pathway*	*KEGG*
*Chemokine signaling pathway*	*KEGG*
*Leukocyte mediated immunity*	*BP*
*Pattern specification process*	*BP*
*Adaptive immune response*	*BP*
*Chemotaxis*	*BP*
*Cytokine production*	*BP*
*Positive T cell selection*	*BP*

From the regulatory network, many biological processes and activated signalling pathways were observed regarding the innate immune response. When this model was constructed with the information obtained from the cytokine chip analysis, we did identify many activated KEGG signalling pathways or biological processes. From this model, several processes were observed in the innate immune response, including antigen presentation, pattern recognition receptor activation, T cell activation, and IgA production, wnt induction of NF-κB activation, cytotoxicity, and apoptosis. Among these processes, GM-CSF was associated with pathogen-associated molecular pattern (PAMP) recognition during the early stages post-infection. KC (CXC1) was found to play an important role in phagosome and chemotaxis. TNF-α is increased rapidly followingthe activation of pattern recognition receptors. Moreover, Mcp-1 was found to be related to wnt, chemotaxis, and leukocyte immunity. These biological processes and signalling pathways were in an activated state in *M. fortis* from the second week post-infection. It was also shown that some cytokines, biological processes, and signalling pathways were up-regulated or down regulated following *S. japonicum* infection of *M. fortis*. (shown in Fig. 2)

**Figure 3:**
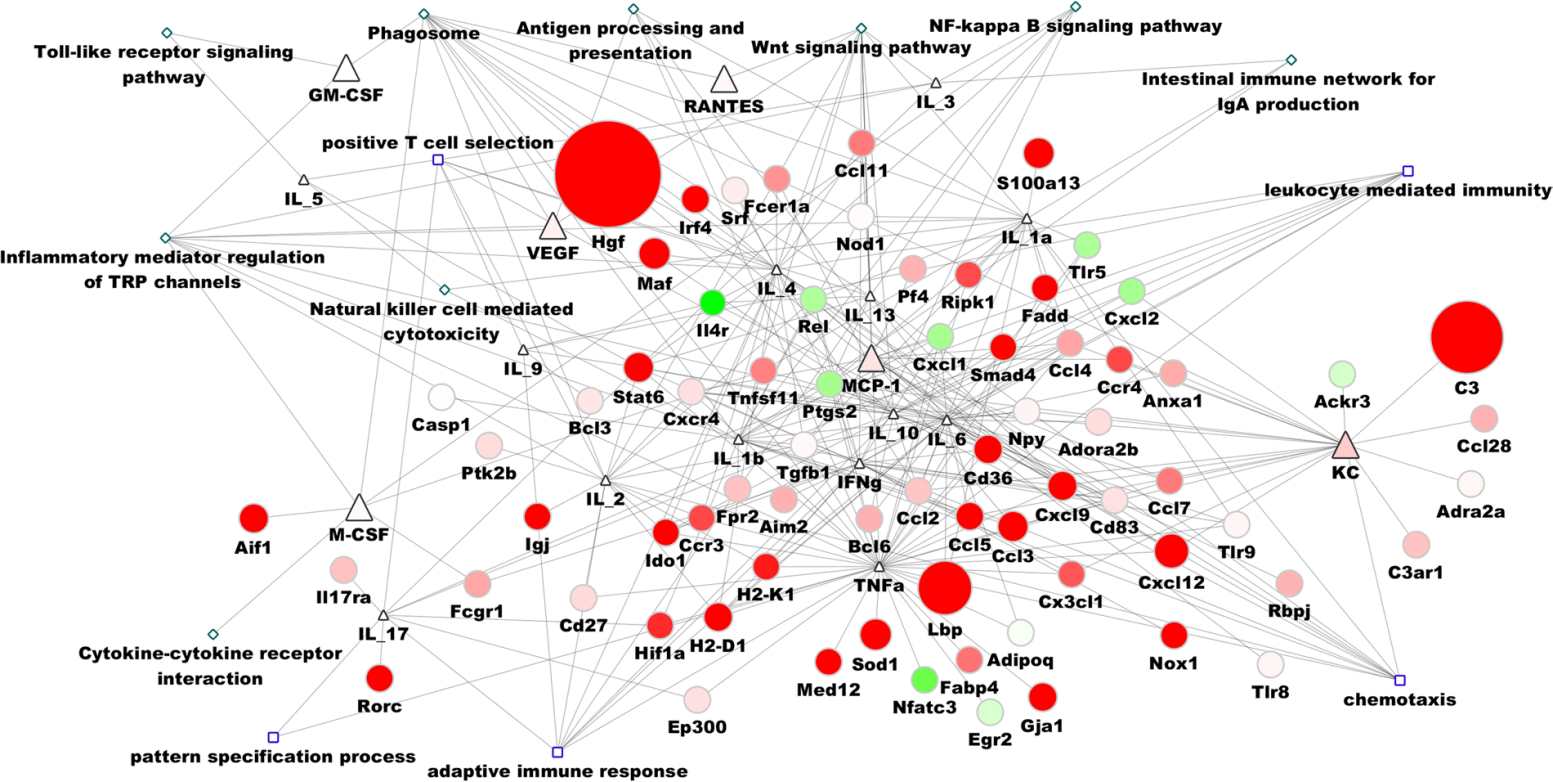
Regulation model of cytokines and transcripts related to cytokine genes. The triangle denotes cytokines; the circle indicates genes that have the potential to interact with cytokines and were detected by RNA-seq; the diamond denotes the KEGG pathway; and the rectangle indicates biological processes. Red represents up-regulation, green indicates down-regulation, and the node size represents the level of fold change in expression. Hgf, C3, and Lbp were substantially increased, which was related to IL-4, KC and TNFα. In contrast, some genes (e.g., CXCL1, CXCL2, and TLR5) decreased significantly.

We found most genes related to cytokines exhibited an upward trend, suggesting the activation of the immune response (shown in Fig. 3). The expression of Hgf, C3, and Lbp several genes were up-regulated to 1000-fold, and were correlated with that of IL-4, KC and TNF-α. This may explain the importance of these cytokines in the immune response against *S. japonicum*. In this model, we also found that some genes exhibited a downward trend (e.g., Ptgs2, Rel, IL4r, CXCL1, CXCL2, TLR5, Nfatc3 and Egr2) in *M. fortis* after infection.

### Analysis of differential metabolites exhibited some metabolomic differences and pathways related to the resistance to *S. japonicum*

The VIP values (threshold value >1) of the OPLS models and the p value (p < 0.05) that resulted from the Student’s tests were used to identify any significantly differentially expressed metabolites. We analysed the differential metabolites between the uninfected (A) and infected (B) *M. fortis* groups. The differential metabolites between the uninfected (C) and infected (D) C57BL/6 mice groups were removed. The remaining data were used to describe the features of infected *M. fortis* (Table 2). It was found that the main differences between the infected and uninfected *M. fortis* were related to HETE, N-Acetylleucine, N-Acetylglucosamine-6-phosphate and 1,4-Methylimidazoleacetic acid. Therefore, these differentially expressed metabolites may be related to the resistance mechanism exhibited by *M. fortis* against *S. japonicum*.

**Table 2:** List of the differentially expressed metabolites between the uninfected (A) and infected (B) *M. fortis* groups.

LC-MS	VIP	mz	Rt	Name	T test	Fold(infected M. fortis/uninfected M.fortis) (B/A)
ESI+	2.192	322.2711	13.75	α-Linolenoyl Ethanolamide	0.002	1.635
	1.890	141.0656	0.86	1,4-Methylimidazoleacetic acid	0.012	1.633
	2.068	280.263	13.69	Linoleamide	0.004	1.416
	1.635	174.1126	6.98	N-Acetylleucine	0.036	1.109
	1.587	622.0764	4.41	UDP-N-acetyl-2-amino-2-deoxy-D-glucuronate	0.043	0.873
	1.551	160.0967	4.80	Acetyl-DL-Valine	0.049	0.777
	2.251	324.2892	13.37	Linoleoyl Ethanolamide	0.001	0.765
	1.913	303.2315	13.75	Eicosapentaenoic Acid	0.010	0.725
	2.052	256.2631	14.09	Palmitic amide	0.005	0.711
	1.782	399.1438	0.81	S-Adenosylmethionine	0.019	0.679
	2.071	177.0384	0.94	Glucuronolactone	0.004	0.642
	1.621	204.1051	7.63	3-Indolebutyric acid	0.038	0.596
	1.604	282.2786	14.27	Oleamide	0.040	0.591
	1.756	330.2634	10.87	4,8 dimethylnonanoylcarnitine	0.022	0.356
ESI−	2.219	333.0592	0.73	2-(beta-D-Glucosyl)-sn-glycerol 3-phosphate	0.003	0.816
	2.448	319.2274	12.78	HETE	0.001	2.380
	1.954	174.0415	0.91	N-acetylaspartate	0.014	1.487
	1.700	300.0431	0.86	N-Acetylglucosamine-6-phosphate	0.039	1.029
	1.784	283.0684	1.86	Xanthosine	0.029	0.677
	1.687	337.2381	11.77	PGA1	0.041	0.659
	1.674	221.0927	3.90	4-aminohippurate	0.043	0.573
	1.919	175.0249	0.90	Ascorbic acid	0.016	0.450
	1.585	130.088	1.49	Leucine	0.058	0.292
	1.564	243.0627	0.90	Uridine	0.062	−0.476
	1.837	567.3507	8.39	PG(22:0)	0.023	−1.615

“foldB/A” indicated the ratio of the average value infected *M. fortis* (group B**)** to uninfected *M. fortis* (group A) (to 2 logs base). A positive value indicates that the concentration of the metabolites in Group B was higher than that of Group A. A negative value denotes that the concentration of the metabolites in Group B was lower than that of Group A.“VIP” indicates the variable importance in the projection; “mz”meant mass-to-charge ratio; “rt” meant retention time.

There were significant differences between the infected *M. fortis* (B) and infected mice groups (D) in both the electrospray ionization (ESI)+ model and the ESI− model. Some of these differences in metabolites were caused by species differences, which were also present between the uninfected *M. fortis* (A) and C57BL/6 mice group (C). After removing the differential metabolites related to species differences, the remaining metabolites were isolated and listed in Table 3. The model analysis suggested that the primary differences were related to quasi-vitamins (e.g., carnitine, stearoylcarnitine, palmitoyl-L-carnitine, and linoleyl carnitine), valine, inosine 5′-monophosphate, sedoheptulose, and indoxylsulfuric acid. These differences in metabolites might be related to the resistance to *S. japonicum*.

**Table 3:** Metabolite differences between the *M. fortis* and C57BL/6 following *S. japonicum* infection.

LC-MS	VIP	mz	rt	Name	T test	Fold(infected Mice/infected M.fortis) (D/B)
ESI+	1.510	285.221	13.16	Retinaldehyde	0.000	1.889
	1.294	284.0987	1.07	Guanosine	0.002	1.262
	1.062	259.095	1.02	Ribothymidine	0.015	1.239
	1.423	255.2316	12.27	cis-9-palmitoleic acid	0.000	1.138
	1.305	227.2003	11.33	Myristoleic acid	0.001	1.126
	1.095	150.0583	0.92	Methionine	0.003	0.553
	1.002	123.0548	1.02	Niacinamide	0.012	−0.188
	1.026	162.1115	0.82	Carnitine	0.033	−0.502
	1.178	130.0496	0.91	Pyroglutamic acid	0.006	−0.515
	1.068	118.0853	0.86	Valine	0.020	−0.889
	1.161	208.0967	7.37	N-Acetyl-D-phenylalanine	0.007	−1.303
	1.006	428.3728	12.53	Stearoylcarnitine	0.026	−1.460
	1.438	400.3416	11.99	Palmitoyl-L-carnitine	0.000	−1.750
	1.415	424.3416	11.77	Linoleyl carnitine	0.000	−2.058
ESI−	1.163	221.0663	0.780	Ethyl glucuronide	0.006	1.160
	1.006	277.2167	13.852	Linolenic Acid	0.030	0.976
	1.004	171.007	0.771	D-Glycerol 1-phosphate	0.031	−0.618
	1.060	193.0359	0.788	D-Glucuronic acid	0.006	−0.685
	1.061	146.0475	0.809	Glutamate	0.010	−0.695
	1.087	178.0507	5.308	Hippuric acid	0.002	−0.792
	1.079	195.0515	0.826	Gluconic acid	0.013	−0.804
	1.166	347.0417	0.920	Inosine 5′-monophosphate	0.003	−1.241
	1.377	128.036	0.946	Pyroglutamic acid	0.000	−1.420
	1.438	368.9892	0.710	Sedoheptulose 1,7-bisphosphate	0.000	−2.046
	1.092	212.0019	5.071	Indoxylsulfuric acid	0.006	−2.193

“fold B/D” indicates the ratio of the average value for the infected mice (Group B) to infected *M. fortis* mice (Group D) (to 2 logs base). A positive value indicates that the concentration of the metabolites in Group B was higher than that of Group D. A negative value denotes that the concentration of the metabolites in Group B was lower than that of Group D. VIP, variable importance in the projection; mz, mass-to-charge ratio; rt, meant retention time.

MetPA was applied to identify the pathways that might be relevant to the resistance exhibited by *M. fortis* against *S. japonicum*. Differential metabolites were found to belong to various metabolic pathways. The pathways associated with the differential metabolites exhibited between Groups A and B, as well as B and D are listed in Table 4, respectively. These pathways included amino sugar and nucleotide sugar metabolism, histidine metabolism, lysine degradation, fatty acid degradation, valine, leucine and isoleucine biosynthesis, biosynthesis of alkaloids derived from histidine, and purine and carbon metabolism.

**Table 4:** Differential metabolite pathways between Group A (uninfected *M. fortis*) and B (infected *M. fortis*), and Group B (infected *M.fortis*) and D (infected C57BL/6).

	Differential metabolites	KEGG	Pathway
Compare group A to B	HETE	NA	NA
	N-Acetylleucine	C02710	NA
	N-Acetylglucosamine-6-phosphate	C00357	Amino sugar and nucleotide sugar metabolism
	1,4-Methylimidazoleacetic acid	C05828	Histidine metabolism
Compare group B to D	carnitine	C00487	Lysine degradation
	stearoylcarnitine	NA	NA
	palmitoyl-L-carnitine	C02990	Fatty acid degradation
	linoleyl carnitine	NA	NA
	valine	C00183	Valine, leucine and isoleucine biosynthesis
	Inosine 5′-monophosphate	C00130	Biosynthesis of alkaloids derived from histidine and purine
	Sedoheptulose	C00447	Carbon metabolism
	Indoxylsulfuric acid	NA	NA

NA, not available.

## Discussion

*M. fortis* is widely distributed throughout schistosomiasis endemic areas of the Dongting Lake and Yangze River basin in China. As early as 1965, reports demonstrated that *S. japonicum* was not able to infect *M. fortis* ^17^, concluding that it was a non-permissive host preventing the adult parasites from producing eggs. In contrast, C57BL/6 mice are a permissive host for *S. japonicum*, in which the parasite can successfully complete its life cycle. While recent research continues to explore this phenomonon, the mechanism remains unclear. In the present study, we used high-through technology to compare the differences between *M. fortis* and C57BL/6 mice following infection with *S. japonicum*, and obtained novel insight into the resistance of *M. fortis* against *S. japonicum*.

After three weeks post-infection, the levels of monokines, Th1, Th2, Th17 cytokines, chemokines, and factors related to angiogenesis in *M. fortis* were significantly higher than that in C57BL/6 mice. This indicated that at this time point, the immune response is activated to a greater extent in *M. fortis* than in C57BL/6 mice. In some hosts that are permissive for *Schistosoma* (e.g., humans and C57BL/6 mice), parasitic infection actively suppresses the immune response^18^.

In *M. fortis*, the level of IL-1β, GM-CSF, M-CSF, MCP-1, and VEGF increased from the second week post-infection, to concentrations significantly higher than that in C57BL/6 mice. Moreover, the genes related to these cytokines, including hepatocyte growth factor (*Hgf*), *C3a*, allograft inflammatory factor-1 (*Aif1*), and lipopolysaccharide-binding protein (*Lbp*), increased significantly (Fig. 3). Hgf has been shown to promote both endotheliocyte and hepatocyte proliferation^19^. In addition, *C3* expression was significantly higher after infection than prior to infection, which was consistent with Zhang’s report^20^. C3 is a type I acute phase protein that can mediate the inflammatory response, and is induced by IL-1β and TNF-α^21^. *Aif-1* is a potent molecule that promotes the expansion and activation of CD4^+^^4^ T cells, plus the promotion of a proinflammatory cytokine milieu^22^. In addition, Lbp is implicated in the binding of LPS to host cells and potentiating its signalling activity. Moreover, in the context of obesity, the overproduction of this protein is a reliable biomarker of chronic inflammation^23^. The high expression of these genes revealed that there was drastic inflammatory response exhibited by *M. fortis* following infection with *S. japonicum*. These cytokines and the activation of related genes promote some KEGG signalling pathways and biological processes, including inflammatory mediator regulation of TRP channels, phagosomes, natural killer cell mediated cytotoxicity, wnt signalling pathway, and NF-κB signalling (Fig. 2).

High levels of chemokines promote innate immune cell activation, cytokine secretion, the activation of inflammatory signalling pathways, and pathogen clearance. Therefore, the natural immunity exhibited by *M. fortis* in response to infection with *S. japonicum* is more active than that in C57BL/6 mice.

In *M. fortis*, the level of IL-2, IL-12, IL-3, IL-4, IL-5, IL-10, IL-17 and IFN-γ were found to be significantly higher than that of C57BL/6 mice. Related genes, including immunoglobulin J polypeptide (*Igj*), indoleamine 2,3-dioxygenase 1 (*Ido1*), signal transducer and activator of transcription 6 (*Stat6*), Myeloid Elf-1-like factor (*Mef*), and others also increased significantly (Fig. 3).

The *Igj* gene encodes the J chain protein of IgM and IgG antibodies. Thus, the increased expression of *IgJ* is indicative of enhanced production of IgM and IgA. Moreover, *Ido1* encodes indoleamine 2,3-dioxygenase, which is the rate-limiting enzyme for tryptophan catabolism. Indoleamine 2,3-dioxygenase (*Ido*) mediated catabolism of tryptophan can modulate the immune system to arrest inflamemation^24^. In addition, high expression of *Ido1* can downregulate the inflammatory response exhibited by *M. fortis* after infection with *S. japonicum*. *Stat6* is downstream of Th2 cytokine signalling^25^, and increased levels of *Stat6* are indicative of a strong T-helper 2 type immune response. *Mef* is an ESTS transcription factor which activates innate immunity-associated genes (e.g., lysozyme [LYZ], human β-defensin 2 [HβD2], and interleukin-8 [IL-8]) in epithelial cells^26^. In the present study, these cytokines and related genes promoted leukocyte-mediated immunity, positive T cell selection, and activation of the adaptive immune response (Fig. 2). Our results demonstrated that adaptive immunity also participated in the response of *M. fortis* against *S. japonicum*. In contrast, the immune response of C57BL/6 mice was suppressed for the first four weeks post-infection.

Metabolomics is a new discipline that seeks to qualitatively and quantitatively analyse all the low molecular weight metabolites of an organism or cell during a particular physiological period^27^, and can provide such information *in vivo*. Following infection with *S. japonicum*, the response exhibited by *M. fortis* significantly differs from that of C57BL/6 mice. Two weeks post-infection, several white nodules were observed in the livers of *M. fortis*, which disappeared by four weeks post-infection. These white nodules consisted of dead schistosomula surrounded by inflammatory cells^15, 28^. In addition, there were no changes that occurred in the livers of C57BL/6 after the first four weeks of infection^15^. Cheng’s report also revealed that from 12 to 18 days post-infection, all cercariae were dying in the livers of *M. fortis* ^29^. Therefore, this significant difference should be reflected in the metabolic reactions of each host. In the present study, we compared the metabolites in the liver tissues of infected and uninfected *M. fortis*, as well as infected *M. fortis* and C57BL/6 mice two weeks post-infection.

An interesting finding was that carnitine and several acylcarnitines (e.g., stearoylcarnitine, palmitoyl-L-carnitine, and linoleyl carnitine) were significantly higher in the livers of infected *M. fortis* than that of infected C57BL/6 mice. Carnitine is a quasi-vitamin which plays an important role on fat metabolism and energy production in mammals^30^. Some reports have shown that carnitines can support the production of CD4^+^ and CD8^+^ T cells during infection^31^. Moreover, it has also been found that L-carnitine supplementation might reduce inflammation in coronary artery disease due to its antioxidant effects^32^. Therefore, high levels of carnitine could be one of the mechanisms by which *M. fortis* mediates protection against *S. japonicum*.

Unsaturated fatty acid and unsaturated fatty amines (e.g., HETE) were also significantly in *M. fortis* than that in C57BL/6 mice following infection with *S. japonicum*. Some studies have reported that higher unsaturated fatty acids affect neutrophils^33^. Neutrophils are very important in the destruction of invading microorganisms and the activation of other immune cells, including B and T lymphocytes. The potent lipoperoxidation agent, 15-HETE, has been identified as a mediator potentially responsible for inducing both IL-1β production and MMP-9 activity^34^. Thus, these studies indicate that HETE might activate neutrophils and promote IL-1β production.

We also found that several amino acids, including N-Acetylleucine, valine, and leucine, were significantly higher in *M. fortis* than in C57BL/6 mice following infection with *S. japonicum*. In addition, leucine-rich repeat sequences are important components of TLR ectodomains^35^. We concluded that following infection, TLRs are significantly expressed, and this expression plays an important role in the schistosomula removal exhibited by *M. fortis*. In the metabolomic pathway analyses, we found that these amino acids were related to the biosynthesis of valine, leucine, and isoleucine, which are branched-chain amino acids (BCAA). Research indicates that BCAA oral supplementation can both improve nutritional status (regarding both protein and energy nutrition), as well as improve the phagocytic function of neutrophils and the NK activity of lymphocytes in cirrhotic patients^36^. Therefore, following *S. japonicum* infection, BCAAs might promote the phagocytic function of neutrophils and NK activity in *M. fortis*, and these functions might play an important role in the removal of schistosomula through TLR signalling.

We identified other compounds (e.g., N-Acetylglucosamine-6-phosphate, Sedoheptulose, 1,4-Methylimidazoleacetic acid, and Indoxylsulfuric acid) were significantly higher in *M. fortis* than in C57BL/6 mice following infection with *S. japonicum*. However, we currently do not know whether these constituents are related to the mechanism of the protective immunity against *S. japonicum* exhibited by *M. fortis*.

The pathways of these different metabolites are involved in histidine metabolism, lysine degradation, valine, leucine, and isoleucine biosynthesis. It was concluded that these metabolism pathways may be related to the resistance mechanism of *M. fortis* against *S. japonicum* as histamine is a critical immune modulator. The histamine H_1_ receptor (H_1_R) mediates proinflammatory effects, including increases in vascular permeability, the expression of adhesion molecules on endothelial cells, and H_2_R-mediated immunosuppressive functions^37^. Moreover, H_4_R regulates the chemotactic responses of leukocytes^37^. Valine, leucine, and isoleucine are BCAAs and several studies have indicated that BCAAs are important during lymphocyte proliferation and dendritic cell maturation^38^. In patients with cirrhosis, BCAAs can increase the number of hepatic lymphocytes and restore the phagocytic activity of neutrophils, as well as the NK activity of lymphocytes^38^. Lysine-modified diversification is the key to the regulation of protein function. Additionally, post-translational modifications (PTMs) of lysine residues have proven to be major regulators of gene expression, protein–protein interactions, as well as protein processing and degradation^39^. Therefore, lysine degradation might be related to the resistance against *S. japonicum* exhibited by *M. fortis*.

In the present study, we used the data obtained from a cytokine protein chip assay, transcriptome, and metabolome together to profile the immune characteristics of *M. fortis* following infected with *S. japonicum*. There were significantly different responses between *M*. *fortis* and mice after infection. Some of these differences may be related to the different number of infected *S. japonicum* cercaria, while most of the different responses were related to the resistance mechanisms of *M. fortis* against *S. japonicum*.

It was found that high levels of cytokines (e.g., IL-1b, IL-3, IL-4, IL-10, IL-17, MCP-1 and VGF) promoted innate and adaptive immune activation, cytokine secretion, inflammation signalling pathway activation, and pathogen clearance. Following infection, the main differences were related to unsaturated fatty acids, quasi-vitamins, amino acids, and other compounds. The differentially expressed metabolic factors were related to histidine metabolism, valine, leucine, and isoleucine biosynthesis, as well as lysine degradation. Such increases may promote the phagocytic function of neutrophils and the NK activity of lymphocytes, which may be related to TLR activation and signalling and lead to the production of IL-1β. Thus, our data may provide a novel foundation that will aid in the further study of the resistance mechanisms employed by *M. fortis* against *S. japonicum*.
